# Modern Sociology

**Published:** 1900-09-29

**Authors:** 


### Modern Sociology.

### THE CRIMINAL ON THE CONTINENT.

The different countries of Europe hold different ideas as to the age when responsibility begins and a person can be regarded as knowing the meaning of his actions. In England the law looks upon everyone over the age of seven as a responsible being; and every child beyond that age can be prosecuted as a criminal. The same age is accepted in Russia and Portugal. In France and Belgium the age is eight, in Italy and Spain it is nine, Norway, Greece, Austria, Denmark, and Holland decline to prosecute a child under 10; and this is the rule also in some of the Swiss cantons. Other cantons exempt a child from personal responsibility up to the age of 12; this is the limit in Germany also. In the Cantons of Vaud and Valais the age is 14, while in Sweden and Finland no prosecution is allowed when the offender is under sixteen years of age. As a matter of fact, however, this difference of opinion as to the age of responsibility is more apparent than real; for in those countries which refuse to regard a child as a criminal until he has attained a fairly mature age, the law still has the power to send him to a reformatory or some institution of that nature. This is practically what is done in England, and the only question is, whether we do the child an injury by labelling him "criminal." As young criminals are everywhere dealt with more leniently than older ones, and even on different principles, this may be regarded as practically only a sentimental wrong.

Thus in Belgium, though an offender is called a criminal from the age of 8, the penal law recognises two distinct periods, and a criminal under 17 is treated differently from one above that age. With the young offender, the judge must take the responsibility of deciding whether or not his moral nature was sufficiently developed for him to understand the nature of his act. If the judge thinks he acted without the knowledge of right and wrong, he is to acquit him, and, after a reprimand, send him home to his parents; but if he thinks that they, through bad example or education, have been the cause of his falling into crime, or if his former environment seem in any way to be morally unwholesome, the judge may either demand from the parent or guardian security for the reform of the child; or, if he has cause to distrust their assurances, may commit the child to a reformatory or charitable institution during his minority, or for any shorter period he may think desirable. He cannot, however, be detained after he is 21. Even if the child acted with knowledge of the moral bearings of his crime, he may, after undergoing a period of imprisonment, be detained under State control till his majority. Sentence of death cannot be pronounced against a criminal under 18 years of age, but imprisonment for life may. Wherever it is possible a fine is substituted for imprisonment, as having to pay for their children's misdeeds is a sure way of making parents keep them more strictly.

The treatment of young criminals in France is very similar to that in Belgium. In the various reformatories a law of 1850 ordains that moral, religious, and trade instruction should be given to the inmates; but an experiment is being tried at an institution at Montesson of abolishing the religious instruction. It will be interesting to see what the result of the change will be. The fact that one of the most successful reformatories for boy offenders is under the care of an Alsatian sisterhood at Frasnes le-Chateau seems to show that religion may still have a place in the making of good citizens. All the reformatories for boys under 12 are under the care of women. In France "conditional conviction" is common, and seems to act very well. The criminal, if a first offender, has sentence pronounced against him, but does not undergo it at the time. If, however, he is again convicted, he has to undergo the sentence for both crimes. Since the law of conditional conviction was adopted in 1891, about 16,000 cases have been dealt with annually, and of those 17 per 1,000 have been reconvicted. By this law a first offender escapes both the stigma and the more or less harmful influences of imprisonment.

In Germany, where children under 12 are not accounted criminals, they may nevertheless be sent to reformatories, or boarded out by the State in private families. No conviction can, however, be recorded against them. Even when they acted with knowledge, and are convicted, the punishment of first offenders is lenient. For slight offences a reprimand only is given, and the offenders are not deprived of their civil rights, nor subjected to police supervision. Bad parents may be deprived entirely of the control of their children, who then become the wards of the State. It may be surmised that bad parents are not unwilling thus to free themselves from parental responsibility. At any rate, crime is increasing faster than population in Germany. The latter increased 20 per cent. between 1888 and 1893; but the former increased 32 per cent.

In Austria criminals under the age of 10 are left entirely to the disciplinary control of their parents; between 10 and 14 their offences are all classed as misdemeanours, and are generally dealt with at home; only in special circumstances are the offenders put under police control. In the latter case the criminal, if under 15, may be sent to a reformatory till he is 20. There are special establishments for beggars and vagrants; and in all these the inmates must be taught a trade, besides receiving moral and religious instruction.

As all the cantons in Switzerland have their own laws, it is impossible to generalise concerning their treatment of their criminal population. Where a reprimand would be looked upon as sufficient punishment in one canton, imprisonment is resorted to in another. But in all it is the rule to keep young offenders as much apart as possible from hardened criminals. Till this was done, it was almost impossible to find situations for those who had been in reformatories, and reconvictions were common. When the criminals were more carefully classified, and treated according to their several degrees of guilt, the reverse became the case.

In Sweden there is an increasing tendency to punish by fine instead of imprisonment. In Denmark, whipping is largely resorted to. Boys may be whipped up to the age of 15, and in certain cases 18, and in that country alone girls are whipped if under the age of 12.

